# Efficacy of 180° Cyclodiode Transscleral Photocoagulation for Refractory Glaucoma

**DOI:** 10.4274/tjo.18559

**Published:** 2018-12-27

**Authors:** Figen Bezci Aygün, Mehmet Cem Mocan, Sibel Kocabeyoğlu, Murat İrkeç

**Affiliations:** 1 Hacettepe University Faculty of Medicine, Department of Opthalmology, Ankara, Turkey

**Keywords:** Cyclophotocoagulation, laser, glaucoma

## Abstract

**Objectives:**

To evaluate the efficacy and safety of transscleral cyclophotocoagulation (TS-CPC) limited to 180° of ciliary body ablation in patients with various forms of refractory glaucoma.

**Materials and Methods:**

Thirty eyes with refractory glaucoma treated with 180° TS-CPC were retrospectively analyzed for intraocular pressure (IOP) reduction and success rates. Patients’ age, gender, type of glaucoma, number of diode laser treatment sessions, postoperative complications, number of hypotensive medications required to control IOP, and best corrected visual acuity (BCVA) were evaluated. The criteria for success were defined as postoperative IOP <21 mmHg or >20% decrease in IOP with or without additional medical treatment.

**Results:**

The mean age of all patients was 51.3±26.9 years (range,1-84 years). The mean postoperative IOP level (23.9±8.5 mmHg) was significantly lower than preoperative IOP (39.2±8.9 mmHg) (p<0.001). The success rate was 66.6% after the first laser treatment and reached 86.7% following repeat laser treatments over an average follow-up period of 22.2±19.9 months. The need for topical hypotensive medications decreased from 2.8±1.0 preoperatively to 2.4±1.3 following TS-CPC (p=0.048). Two patients (6.6%) had a one-line decrease in their BCVA following TS-CPC. Transient hypotony and hyphema developed in 4 patients (13.3%). Total laser energy delivered did not correlate with either preoperative (rho=0.10; p=0.594) or postoperative IOP (rho=0.21; p=0.260).

**Conclusion:**

TS-CPC limited to 180° of ciliary body ablation is associated with a reasonable success rate and low incidence of adverse effects in patients with refractory glaucoma.

## Introduction

Destruction of the ciliary body using various methods is an option for the management of refractory glaucoma when conventional medical and or surgical modalities fail to adequately control intraocular pressure (IOP).^1,2,3^ Transscleral cyclophotocoagulation (TS-CPC) is currently a widely employed method of ciliary body ablation that reduces aqueous humor formation by destroying the ciliary body and ciliary epithelium using a continuous diode laser energy source.^4^ In its most common form, TS-CPC employs an 810 nm semiconductor diode laser, the energy of which has been shown to be absorbed by melanin in the ciliary epithelium. During this process, the surrounding tissues including the ciliary body and its vascular supply are destroyed by the resultant thermal energy transfer.^5^

TS-CPC has been established as an alternative therapeutic modality to tube shunt or augmented trabeculectomy for refractory glaucoma, especially in eyes with poor visual potential.^6^ Reported IOP reduction for this procedure has varied between 12% and 66% in different studies.^1,2,7,8^ The extent of response to TS-CPC differs depending on the underlying glaucoma, with higher success rates attained in primary open-angle glaucoma (POAG), neovascular glaucoma (NVG), and inflammatory glaucomas compared with congenital, juvenile, and traumatic glaucoma, and glaucoma following cataract surgery (i.e., aphakic glaucoma).^9^

Despite the widespread use of TS-CPC, published reports reveal that treatment centers vary significantly with respect to their laser energy delivery and postoperative management schemes. Extent of ciliary body treatment, total amount of delivered energy, and use of postoperative anti-inflammatory medications are likely to impact overall efficacy and safety outcomes.^2,3,6^ It has been shown that more extensive treatment of the ciliary body and total energy delivery of >80 J are associated with unfavorable outcomes such as hypotony and phthisis.^3^ Analysis of data from standardized treatment protocols may help refine the TS-CPC technique with potentially improved outcomes and reduced adverse effects. Thus, the purpose of the current study was to evaluate the efficacy and safety of TS-CPC limited to 180° of ciliary body ablation in patients with various forms of refractory glaucoma.

## Materials and Methods

This was a retrospective study undertaken at a single academic institution. The study adhered to the tenets of the Declaration of Helsinki and was undertaken with institutional review board approval from the Institutional Ethics Committee for Human Research.

The clinical records of patients who were diagnosed with glaucoma and underwent TS-CPC between 2006 and 2015 were reviewed. Parameters for the study included type of glaucoma, number and type of anti-glaucoma medications, visual acuity, slit-lamp biomicroscopic examination findings, and IOP measurements. Goldman applanation tonometry (GAT) and Perkins tonometry were used to obtain IOP measurements in adults and children, respectively. Data related to pre- and post-treatment IOP levels, complications, and the need for topical glaucoma medications were extracted for all patients. Treatment details included number of sessions and number of diode laser spots applied per session.

The entire treatment protocol was performed under operating room conditions. The procedures were performed under either general anesthesia or retrobulbar anesthesia (3 cc 2% lidocaine, 2 cc 0.5% bupivacaine hydrochloride). TS-CPC was performed using a transscleral contact fiberoptic G probe attached to the OcuLight SLx semiconductors laser unit (Iris Medical, Mountain View, CA, USA). Laser duration was set at 1,500 ms in all patients. The probe was placed approximately 1.5 mm posterior to the limbus. For each session, an area of 180° around the limbus was treated. The laser power was initially set at 1,500 mW and was increased by 150 mW until the “pop” sound was heard, at which time power was decreased until no audible pops were heard, up to a maximum level of 3,000 mW. Transillumination was used to identify the ciliary body position if uncertain, as in congenital glaucoma or in those with a previous history of intraocular surgery. The laser spots were not applied at the 3 and 9 o’clock positions to avoid potential damage to the ciliary vessels and nerve. Following the procedure, topical anti-inflammatory therapy was initiated. Hypotony was defined as an IOP level of ≤5 mmHg. Patients were followed up at 1 day, 1 week, and 1 month after the procedure and every 3 months thereafter. Patients were not included if they were not followed for at least 3 months. The total cyclodiode energy (J) was calculated by multiplying the number of laser burns by the duration (s) of each burn by the power (W) of each burn. If more than one session was applied, cumulative energy amounts from all sessions were calculated.

The criteria for success included IOP <21 mmHg with or without additional treatment, or a >20% decrease in IOP. Patients who were found to have inadequate IOP control at the 1-month postoperative visit or beyond received additional medical or surgical treatments.

### Statistical Analysis

Microsoft Excel software (Office Excel 2013, Microsoft Corporation, Redmond, WA, USA) were used for data collection. Descriptive data were presented as mean ± SD. Data analysis was performed using SPSS 22.0 (Statistical Package for Social Sciences; SPSS Inc. IBM, Armonk, NY) software package. Independent samples t-test was used for comparisons or Wilcoxon signed rank test was used where necessary. The Spearman’s Rho test was used in correlation analyses. A p value of <0.05 was accepted as statistically significant.

## Results

Thirty eyes of 30 patients (16 males, 14 females) were included in this study. The mean age of all patients was 51.3±26.9 years (range=1-84 years). Types of glaucoma included in the study are summarized in Table 1. Neovascular glaucoma was the most common indication for TS-CPC (30.0%). There was no significant difference in preoperative IOP values between the groups (p=0.282). In total, 66.8% of patients had secondary glaucoma.

The mean postoperative follow-up period was 22.2±19.9 months (range=3-84 months) after the first cyclodiode laser application. There was a significant (30.9%) IOP reduction at the 3-month post-treatment visit (27.1±8.1 mmHg) compared to baseline (39.2±8.9 mmHg) (p<0.001). Successful results were obtained in 20 patients (66.6%) at the 1-month post-laser control. Postoperatively, 80% of patients who underwent laser treatment still required anti-glaucoma eye drops for IOP control. Sustained IOP control for a duration of at least 12 months was achieved in 14 patients (46.6%).

A second TS-CPC session was required in 16 patients (53.3%) whose IOP could not be adequately controlled following a mean interval of 5.4±3.0 months from the initial TS-CPC procedure. In this subset of patients, postoperative success was attained in 50% of cases. Following the second treatment session, an overall successful outcome was achieved in 73.3% of study eyes. Four (13.3%) patients needed a third laser session within 12 months. Repeat treatments were performed in eyes which had congenital, juvenile, and neovascular glaucoma (Table 2). During the course of follow-up, one of the patients refused a repeat laser treatment and two were lost to follow-up after the first 3 months. The fourth patient with congenital glaucoma did not achieve a successful outcome after the third diode laser application. Overall, a successful IOP reduction was attained in 86.7% of the study patients.

At final evaluation, the mean postoperative IOP level (23.9±8.5 mmHg) was significantly lower than the mean preoperative IOP (39.2±8.9 mmHg) (p<0.001). In patients with successful outcomes, the mean percentage of IOP reduction was 43.8±17.3%, 46.7±17.4%, and 43.9±6.3% respectively after the first, second, and third cyclodiode laser treatment sessions. The number of active medications decreased from 2.8±1.0 to 2.4±1.3 following treatment (p=0.048). Mean IOP levels, number of glaucoma medications, and mean amount of total energy are presented in Table 1. Short-term hypotony lasting for <2 weeks was observed in 2 patients (6.6%), and 1 patient (3.3%) with neovascular glaucoma had transient hyphema. Hyphema resolved within 1 month following treatment with observation. In another patient, hyphema and phthisis bulbi were noted 3 years after the procedure when the patient returned for a follow-up visit, but not within the 3-month postoperative follow-up interval. There were no cases of retinal detachment, uveitis, or sympathetic ophthalmia. Visual acuity was <20/400 in 28 patients (93.3%). In the other 2 patients (6.6%), VA decreased by 1 line following TS-CPC. Four patients (13.3%) had no light perception prior to treatment and diode laser was performed for ocular pain control.

The mean age of the patients who required repeated (≥2) diode laser applications (44.2±30.9 years) was not significantly different than those who received a single laser application (59.5±19.4 years) (p=0.112).

The mean number of treatment sessions was 1.6±0.7. The mean laser energy delivered per treatment session was 35.4±16.6 J. The cumulative laser energy delivered after all treatment sessions was 58.9±34.7 J per patient. There was no correlation between the amount of laser energy delivered per session and preoperative IOP level (rho=0.27; p=0.142), postoperative IOP level (rho=0.07; p=0.698), or patient age (rho=0.35; p=0.527). The number of sessions was positively correlated with total delivered energy (rho=0.54; p=0.002) and negatively correlated with postoperative number of glaucoma medications (rho=-0.39; p=0.030). Total amount of energy delivered did not correlate with preoperative IOP (rho=0.10; p=0.594), postoperative IOP (rho=0.21; p=0.260), number of preoperative (rho=-0.08; p=0.662) or postoperative (rho=-0.07; p=0.695) glaucoma medications, nor with mean IOP reduction (rho=-0.09; p=0.626) following diode laser treatment.

## Discussion

Cyclodiode photocoagulation is frequently employed at a clinical stage where adequate IOP control is not achieved despite repeated surgical interventions and maximum medical therapy in glaucoma patients with severely compromised visual function. Despite its proven efficacy, the utility of this treatment modality in achieving IOP control has often been eclipsed by unpredictable responses as well as its potential adverse effects of persistent hypotony and the dreaded complication of phthisis bulbi. The findings of the current study reveal that limiting the extent of laser treatment to 180 degrees is associated with a low incidence of adverse effects and appears to be relatively safe, with about 67% of eyes achieving satisfactory IOP lowering after a single treatment session.

In a previous study using 270° ciliary body ablation on 27 eyes, the cumulative probability of success of TS-CPC was 72% in the first and 52% in the second postoperative year based on success criteria similar to those used in the current study.^10^ Another study by Mistlberger et al.^1^ found the success rate of this procedure with a single treatment was 66.7% at 1 year and 49.7% after 2 years. Although long-term follow-up data for patients treated with a single session were not available, we observed that the 1-month success rate of 66.6% fell to 46.6% at the end of the first year. Thus, the findings of the current study as well as those from other studies indicate a time-dependent loss of IOP control following TS-CPC.

In our study, the cumulative percentage of eyes successfully treated with a single treatment session was found to be 46.6% at the end of 1 year, with repeat laser sessions resulting in a higher success rate (86.7%) at the end of an average of 22.2 months of follow-up. Similar results were obtained in another study that had a 58.9% success rate after the first laser session and a significant increase to 81.3% success with a second treatment session.^2^ Overall, data available from previous studies support the beneficial effect of repeat treatment sessions for IOP control in patients undergoing TS-CPC. Our study findings show that even 180-degree repeat laser treatment is able to provide additional benefit in refractory glaucoma cases.

In a study by Murphy et al.^2^ in which TS-CPC was applied over 270° of the ciliary body, the IOP reduction rate was found to be 52.6% in the first 7 months of follow-up. In a similar study, Singh et al.^11^ found the reduction in IOP to be 58.5% at 9 months post-treatment. In the current study, the IOP reduction rate was 30.9% and 39.0% at 3 and 22.2 months, respectively. One plausible explanation for the limited IOP reduction observed may be related to the lesser extent of ciliary ablation (180° versus 270°) performed in the current study.

A second laser session was required in 53.3% of patients in our study. This group consisted predominantly of cases of juvenile glaucoma, congenital glaucoma, and neovascular glaucoma. Although we did not observe a greater need for repeat interventions among younger patients, a previous study by Threlkeld and Johnson^12^ showed an increased need for multiple laser treatment in young patients, which the authors believed was potentially attributable to a higher level of baseline aqueous humor production and more vigorous healing response in the younger population.

A previous study by Threlkeld and Johnson^12^ demonstrated a significant IOP reduction following TS-CPC in patients with neovascular glaucoma and emphasized caution for post-operative hypotony. In our study, two patients who developed hypotony had either neovascular or juvenile glaucoma. In both patients, preoperative IOP values and mean total energy delivered were higher than the total group average (Table 1). Our findings lend support to the hypothesis that high IOP may cause ciliary body ischemia and increase the risk of hypotony, as was also pointed out by Murphy et al.^2^

Oguri et al.^13^ compared the Nd:YAG laser and diode laser techniques in neovascular glaucoma and found diode laser to be more effective in IOP control. Mistlberger et al.^1^ found that neovascular glaucoma responded well to TS-CPC in their study, but was associated with a higher rate of hyphema and phthisis bulbi. In our study, the mean preoperative and postoperative IOP levels in patients with neovascular glaucoma were 44.1±9.2 mmHg and 20.5±6.1 mmHg, respectively (p=0.008). Despite an overall adequate IOP reduction in these patients, complications including hypotony and hyphema occurred in 2 cases (6.6%). Thus, it is recommended that hypotony may occur infrequently even with 180 degrees of ciliary body ablation and that vigilant IOP monitoring should be practiced even in patients who undergo limited (i.e., 180°) laser ablation.

Schlote et al.^9^ suggested that the success rate of TS-CPC may be related to the type of glaucoma. The authors reported a high success rate in patients with POAG (89.5%), NVG (86.8%), and inflammatory glaucoma (75%) and a lower success rate in traumatic (57.1%), aphakic (57.1%), and congenital or juvenile (62.5%) glaucoma.^9^ In our study, patients with NVG achieved a success rate of 55% with one and 100% with repeat laser treatment. All cases with POAG (n=3) achieved long-lasting IOP control with a single treatment session. On the other hand, the success rate was unsatisfactory for patients with congenital and juvenile open-angle glaucoma, who required repeat laser sessions and ultimately were unable to achieve adequate IOP control (Table 2). Poor response to treatment in the congenital and juvenile groups may be due to the good ciliary body epithelial healing response in this younger patient population.^12^

In 6.6% of our patients, visual acuity was decreased by 1 Snellen line due to cyclodiode application. In the study conducted by Mistlberger et al.,^1^ reduction of visual acuity by ≥2 Snellen lines was reported in 18.7% of patients. In another study by Spencer and Vernon,^14^ at least 2 lines of visual acuity loss was detected in 32% of the patients. The reason for this difference may be the already low preoperative visual acuity of the patients included in the current study as well as the small number of patients in the study, which may preclude direct comparisons across studies.

IOP control can be achieved with the use of multiple glaucoma medications in refractory cases of glaucoma. The total number of active molecules used by the study patients in the current study prior to TS-CPC application was 2.8±1.0. Following diode treatment(s), this number decreased to 2.4±1.3 (p=0.042). Four patients (13%) could be taken completely off topical hypotensive medications following TS-CPC. Thus, the findings of our study are in agreement with those of previous reports showing a reduced need for glaucoma drops following laser treatment, but the need to continue medical treatment in the majority of patients postoperatively.^6,12,15^

As there is no standardization of the magnitude and extent of laser energy used during TS-CPC, it is difficult to make comparisons between different studies. Certain studies reveal a correlation between total amount of energy and success rate^6,16^ while others fail to detect this association.^7,9,11^ In our study, no association was found between energy delivered and postoperative IOP. This discrepancy may stem from the fact that there is really no anatomic landmark during TS-CPC to ensure exact placement of the probe tip over the ciliary body and no method available to assess the energy delivered to the ciliary body tissue.

### Study Limitations

The limitations of the study include the small sample size and its retrospective nature. However, the strength of our study is the availability of data from patients who underwent a homogenous TS-CPC technique; 180° of ciliary body ablation was documented in all cases.

## Conclusion

In conclusion, a reasonable IOP reduction can be achieved with 180° cyclodiode photocoagulation in patients with refractory glaucoma. As such, 180° TS-CPC appears to be a safe alternative to the 360° circumferential technique with very low risk of adverse effects for glaucoma patients with a history of previous failed glaucoma surgeries and limited visual potential. The possibility of repeat treatment sessions and the likely need for postoperative glaucoma medications should be discussed with patients prior to TS-CPC to ensure realistic post-surgery expectations.

## Figures and Tables

**Table 1:**
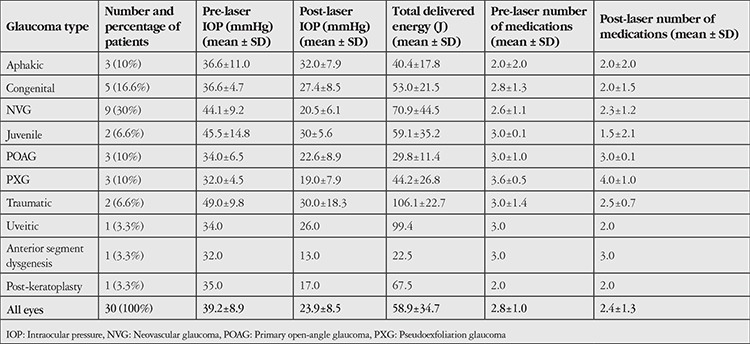
Clinical parameters of the study subjects

**Table 2:**
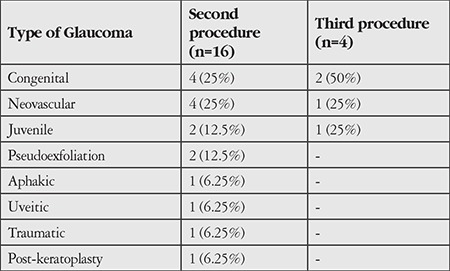
Evaluation of need for repeat cyclophotocoagulation application based on glaucoma subtypes

## References

[1] Mistlberger A, Liebmann JM, Tschiderer H, Ritch R, Ruckhofer J, Grabner G (2001). Diode laser transscleral cyclophotocoagulation for refractory glaucoma. J Glaucoma..

[2] Murphy CC, Burnett CA, Spry PG, Broadway DC, Diamond JP (2003). A two centre study of the dose-response relation for transscleral diode laser cyclophotocoagulation in refractory glaucoma. Br J Ophthalmol.

[3] Ishida K (2013). Update on results and complications of cyclophotocoagulation. Curr Opin Ophthalmol..

[4] Mandal S, Gadia R, Ashar J (2009). Diode Laser Cyclophotocoagulation. Journal of Current Glaucoma Practice..

[5] Pantcheva MB, Kahook MY, Schuman JS, Noecker RJ (2007). Comparison of acute structural and histopathological changes in human autopsy eyes after endoscopic cyclophotocoagulation and trans-scleral cyclophotocoagulation. Br J Ophthalmol..

[6] Zhekov I, Janjua R, Shahid H, Sarkies N, Martin KR, White AJ (2013). A retrospective analysis of long-term outcomes following a single episode of transscleral cyclodiode laser treatment in patients with glaucoma. BMJ Open..

[7] Egbert PR, Fiadoyor S, Budenz DL, Dadzie P, Byrd S (2001). Diode laser transscleral cyclophotocoagulation as a primary surgical treatment for primary open-angle glaucoma. Arch Ophthalmol..

[8] Gupta V, Agarwal HC (2000). Contact trans-scleral diode laser cyclophotocoagulation treatment for refractory glaucomas in the Indian population. Indian J Ophthalmol.

[9] Schlote T, Derse M, Rassmann K, Nicaeus T, Dietz K, Thiel HJ (2001). Efficacy and safety of contact transscleral diode laser cyclophotocoagulation for advanced glaucoma. J Glaucoma..

[10] Kosoko O, Gaasterland DE, Pollack IP, Enger CL (1996). Long-term outcome of initial ciliary ablation with contact diode laser transscleral cyclophotocoagulation for severe glaucoma. The Diode Laser Ciliary Ablation Study Group. Ophthalmology..

[11] Singh K, Jain D, Veerwal V (2017). Diode laser cyclophotocoagulation in Indian eyes: efficacy and safety. Int Ophthalmol..

[12] Threlkeld AB, Johnson MH (1999). Contact transscleral diode cyclophotocoagulation for refractory glaucoma. J Glaucoma..

[13] Oguri A, Takahashi E, Tomita G, Yamamoto T, Jikihara S, Kitazawa Y (1998). Transscleral cyclophotocoagulation with the diode laser for neovascular glaucoma. Ophthalmic Surg Lasers..

[14] Spencer AF, Vernon SA (1999). “Cyclodiode”: results of a standard protocol. Br J Ophthalmol..

[15] Bitirgen G, Okka M, Bozkurt B, Doğru İ, Kerimoğlu H, Turgut Öztürk B, Kamış Ü (2012). Transscleral diode laser cyclophotocoagulation in refractory glaucoma. Turk J Ophthalmol..

[16] Tzamalis A, Pham DT, Wirbelauer C (2011). Diode laser cyclophotocoagulation versus cyclocryotherapy in the treatment of refractory glaucoma. Eur J Ophthalmol..

